# Impact of post-release community mental health and disability support on reincarceration for prisoners with intellectual disability and serious mental illness in NSW, Australia

**DOI:** 10.1192/bjo.2023.9

**Published:** 2023-02-27

**Authors:** Julian Trofimovs, Leanne Dowse, Preeyaporn Srasuebkul, Julian N. Trollor

**Affiliations:** Department of Developmental Disability Neuropsychiatry, Discipline of Psychiatry and Mental Health, University of New South Wales, Sydney, Australia; University of New South Wales School of Social Sciences, Sydney, Australia; Department of Developmental Disability Neuropsychiatry, Discipline of Psychiatry and Mental Health, University of New South Wales, Sydney, Australia; and Centre for Healthy Brain Ageing, Discipline of Psychiatry and Mental Health, University of New South Wales, Sydney, Australia

**Keywords:** Intellectual disability, mental health, data linkage, custody, recidivism

## Abstract

**Background:**

Prisoners with an intellectual disability are overrepresented in custody and more likely to reoffend and be reincarcerated compared with the general prison population. Although prisoners with intellectual disability have many of the same risk factors for recidivism as the general prison population, the high rates of mental illness experienced by this group are key drivers of recidivism.

**Aims:**

We aimed to assess the impact of provision of post-release disability and community mental health support on rates of reincarceration in a cohort with identified intellectual disability and serious mental illness diagnosis.

**Method:**

We conducted a historical cohort study using linked administrative data-sets, including data on hospital admissions, community mental health, disability support and corrections custody in New South Wales, Australia (*n* = 484). To assess the time to return to adult custody, we used survival analysis on multiple failure-time data.

**Results:**

Over the median follow-up period of 7.4 years, 73.7% (357) received community mental health support, 19.8% (96) received disability support and 18.6% (85) received a combination of supports during a post-release period from prison. Lower hazards of reincarceration in a post-release period were associated with receipt of community mental health support (hazard ratio [HR] = 0.58, CI 0.49–0.69, *P* < 0.001), or a combination of community mental health and disability support (HR = 0.46, CI 0.34–0.61, *P* < 0.001).

**Conclusions:**

High rates of reincarceration for prisoners with intellectual disability and history of serious mental illness may be modifiable by provision of appropriate mental health and disability supports.

Prisoners with intellectual disability experience higher rates of mental illness compared with the general prison population;^1^ this is thought to be a significant factor driving reincarceration.^2^ Intellectual disability refers to the concurrent presence of intellectual deficits operationalised as an IQ score below 70, paired with significant limitations in adaptive functioning, which appear during the developmental period.^3^ Estimates of the prevalence of intellectual disability in the general prison population vary widely.^4^ Methodological challenges make accurate ascertainment of intellectual disability prevalence in prison difficult; however, numerous studies report an overrepresentation of intellectual disability in prisons, both internationally^5–7^ and in Australia.^8^

Prisoners with intellectual disability are at greater risk of reoffending^9^ and are more likely to be reincarcerated^8,10^ than the general prison population. Although the specific reasons for this are unclear, numerous studies have identified that prisoners with intellectual disability share risk factors for reoffending with the general prison population,^11^ together with further risk factors unique to their disability, including communication and social skills difficulties, poor judgement or impulse control, and suggestibility and exploitability.^14,15^

Although studies of the provision of post-release support are limited,^12^ a number of studies indicate that post-release support appears to address immediate risk factors for reoffending, including support with accommodation, income, and employment, as well as linking individuals to community support services aimed at addressing longer-term risk factors for recidivism such as alcohol and drug dependence, daily living and community living skills and, in particular, mental illness.^13,14^ To address the overrepresentation in prison and the higher rates of reincarceration of people with intellectual disability, more research is required to assess the impact of post-release disability and mental health support on rates of reincarceration. We aimed to explore a linked administrative data-set, including corrections, hospital admissions, disability and community mental health support services, to assess the impact that provision of post-release disability and community mental health support has on rates of reincarceration in a cohort with identified intellectual disability and serious mental illness (SMI) diagnosis.

## Method

This retrospective cohort study used data drawn from a broader linkage infrastructure^15^ examining the epidemiological profiles of people with neuropsychiatric disorders in New South Wales (NSW), Australia. The data-set contains a near-whole-population sample of individuals with intellectual disability in NSW (*n* = 92 542), approximately 1.13% of the NSW population in calendar year 2015. The linkage contains data from disability services, ambulatory mental health services, targeted specialist support services in public schools, Corrective Services NSW, NSW Ombudsman and NSW Public Guardian services; and patient data from hospital admissions and emergency department presentations in NSW between 1 January 1994 and 30 June 2016.

### Data

#### NSW offender integrated management system

Custody data were from the NSW Offender Integrated Management System (OIMS), containing information relating to prisoner custodial episodes, location and transfer history, classification, security and self-harm behaviour in custody for the period 1 January 1994 to 31 May 2016.

#### NSW Mental Health Ambulatory Data Collection

Community mental health treatment data were sourced from the Mental Health Ambulatory Data Collection (MH-AMB), containing information on NSW specialist ambulatory mental health services for the financial years 1 January 2001 to 31 December 2015. The MH-AMB provides data regarding clinical contact with community mental health services such as mental health day care programmes and psychiatric out-patient and outreach services for non-admitted patients with psychosis.

#### NSW Admitted Patient Data Collection

The Admitted Patient Data Collection (APDC) records all admitted-patient services provided by NSW public hospitals, public psychiatric hospitals, public multi-purpose services, private hospitals and private day procedures centres. The APDC contains admissions data for the financial years 1 July 2001 to 30 June 2016.

#### NSW Disability Services Minimum Data Set

The Disability Services Minimum Data Set (DSMDS) is a de-identified data-set which collates information about people receiving disability services in NSW, including the nature of the disability and the services provided to persons with a disability. Disability services data were available for the financial years 1 July 2005 to 30 June 2015.

#### Population

**Fig. 1:**
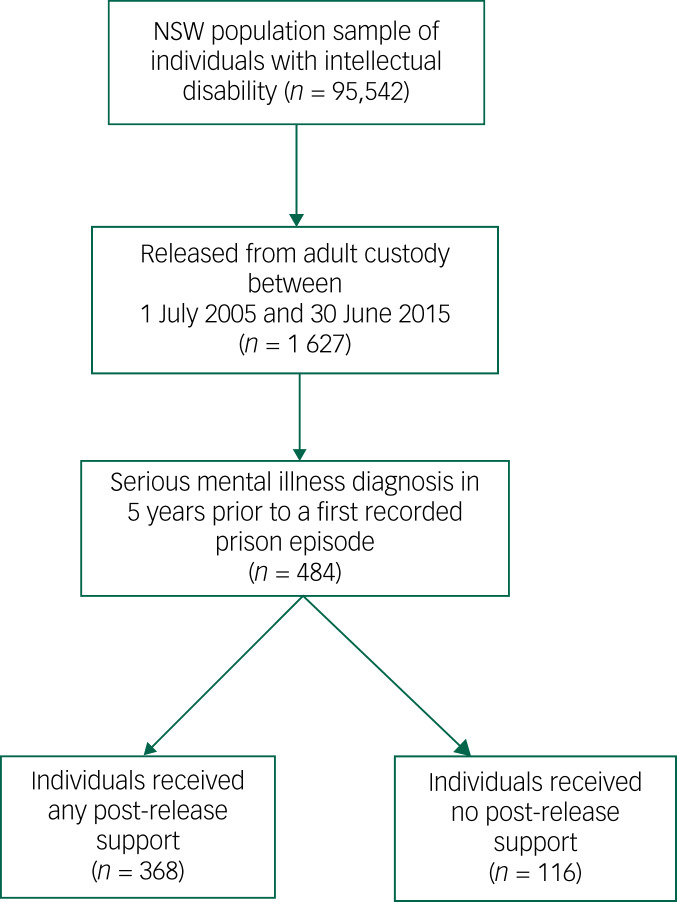
Flow chart of study population.

The study's sample included all individuals released from an adult custodial episode (remand or sentenced) over the study period 1 July 2005 to 30 June 2015 who had both a recorded intellectual disability and SMI diagnosis in the linked data-set (Fig. 1).

#### Intellectual disability diagnosis

No single registry in NSW contains information regarding all individuals diagnosed with intellectual disability in NSW. Thus, the presence of intellectual disability for an individual was determined by diagnostic labels contained in a range of data-sets. Individuals were identified as having intellectual disability across one or more of the linked data-sets based on the presence of either a DSM-IV or an ICD-10 diagnosis of intellectual disability.^15^

#### SMI diagnosis

People with SMI were identified on the basis of ICD-10 diagnoses of bipolar affective disorder, mania, schizophrenia, schizoaffective disorder or other psychotic disorders (ICD-10 codes F20*–25* and F28*–31).^16^ Individuals that had at least one hospital admission in the 5 years prior to a first recorded prison episode in the study period, where a SMI diagnosis was recorded, were included in the study sample.

### Variables

#### Outcome variable

The outcome variable was time until next reincarceration. This was calculated as the number of days from custody release date (remand or sentenced) until the next custodial episode start date (remand or sentenced). Thus, the observation period during which an individual could be reincarcerated started at the date on which the person was released from custody and finished when the person returned to custody or died, or at the end of the study period. An individual could have multiple custodial episodes and periods of release during the study period.

#### Community mental health support variable

To investigate the effect of mental health support immediately following release from custody, post-release community mental health support was defined as the individual having received one or more community mental health clinical services as recorded in the MH-AMB data-set at any point in the first 6 months post-release from adult custody. Mental health service contact was determined following a manual review of the activity codes relating to contacts with community mental health services recorded in the MH-AMB data-set to confirm that the visit was clinical in nature (as opposed to administrative). A community mental health contact was indicated through a binary flag. The binary flag had a value of ‘0’ before receiving any services and ‘1’ after the service. Community mental health support was a time-variant variable in the survival model, with the values checked every year in the study period.

#### Disability support

Provision of disability support was identified by a recorded entry in the linked disability support DSMDS data-set. Access to disability support in NSW requires that the impairment is likely to be permanent, affects the person's capacity for social and economic participation, and substantially reduces their ability to take part effectively in activities (i.e. communication, social interaction, learning, mobility, self-care or self-management) or perform tasks or actions without assistance. The main supports included accommodation, community support, community access and respite. Owing to the aggregated nature of the disability data-set, determination of the nature of the support received was not possible; it could only be determined that support (in one form or multiple forms) was received during a given financial year. Those indicated as having received disability support for the financial year in which they were released from adult custody were assumed to have received a disability service post-release. This was indicated through a binary flag, where the binary flag had a value of ‘0’ before receiving any services and ‘1’ after the service. Disability support was a time-variant variable in the survival model, with the values checked every year in the study period.

#### Criminal justice variables

Criminal justice variables included in the analysis were drawn from the linked OIMS data-sets. These included: length of custodial episode in days (time variant); and number of prior adult custodial episodes (sentenced or remand) categorised as 0 episodes (reference group), 1–5 episodes, 5–10 episodes or 10 + episodes.

#### Substance use disorder

Alcohol or drug substance use was identified through any recorded mental and behavioural disorders due to psychoactive substance use (F10-F19) diagnosis in the 5 years prior to first adult custody episode in the admitted patient data-set.

#### Demographic variables

A range of demographic variables were included in the analysis, including gender (coded 1 for male, 0 for female), age at commencement of study (coded as 18–24, 25–29, 30–39, 40–49 or 50+ years), country of birth (coded as 1 for Australia and 0 for other) and Indigenous status (coded 1 for yes and 0 for no). However, although Indigenous status was included as a control variable in this study's model, ethics approval from the Aboriginal Health and Medical Research Council only related to reporting Indigenous results as a control variable. This limited our ability to report on Indigenous status in this study. The Index of Relative Socio-economic Disadvantage, which summarises information about economic and social conditions including income, employment, qualifications and occupation within an area,^17^ was also used. Statistical area was also used to code remoteness (major cities, inner regional, outer regional, remote or very remote), defined according to the Australian Bureau of Statistics coding of area remoteness.^18^ A count of mental health hospital admissions in the 5 years prior to first incarceration was also included in the analysis.

### Statistical analysis

Descriptive statistics for demographic characteristics, custodial-related variables, and mental and behavioural diagnoses were reported. Low cell counts in aggregated data tables (cells with a count of <10) were omitted from the table.

To assess the time to return to adult custody, we used survival analysis on multiple failure-time data. Multiple failure-time data arise from time-to-occurrence studies where two or more events (failures) occur for the same subject. In studies using multiple failure-time data, failure times are correlated within cluster (subject or group), violating the independence of failure times assumption required in traditional survival analysis. To address this, a frailty model was used.^19^ The use of the frailty model in this context enabled control for unmeasured individual differences in the likelihood of failure (reincarceration). A Cox proportional hazard regression model was used to determine the factors associated with return to custody. Proportional hazards were checked using statistical tests and graphical diagnostics based on the scaled Schoenfeld residuals. To assess whether categorical variables (with more than two levels) improved model fit, we used a Wald test. Variables that failed to add value to the model could be omitted without affecting the model in any meaningful way.

All analysis and figures were undertaken using freely available software packages run in the free software statistical environment R (http://cran.r-project.org). Survival analysis was conducted using the R package ‘survival’.^20^

### Ethics

The study was approved by the NSW Population and Health Services Committee (AU RED reference: HREC/13/CIPHS/7; Cancer Institute NSW reference number: 2013/02/446, sub-study reference number: 2019UMB1001).

## Results

### Descriptive statistics

Table 1 summarises the characteristics of the cohort. Of those identified as having intellectual disability and a past SMI (*n* = 484), the majority were male (80.8%), 18 to 24 years old at the start of the study period (35.7%), resided in major cities (63.8%) and had a mean imprisonment period of 187.8 days (s.d. 392.6), and the majority had a diagnosed substance use disorder diagnosis (80.9%). During a post-release from custody period, 73.7% (357) received community mental health support, 19.8% (96) received disability support and 18.6% (85) received a combination of mental health and disability support. A total of 116 (24.7%) individuals received neither community mental health nor disability support at any point in the study period. In the 5 year period prior to a first incarceration episode, the study cohort had a median of four (range 1–89) hospital admissions where a SMI diagnosis was recorded.

**Table 1:** Study cohort characteristics by community mental health support provision post-release

	Study cohort (*n* = 484)
Male (%)	391 (80.8)
Female (%)	93 (19.2)
Country of birth – Australia (%)	442 (91.3)
Mean custody length, days (s.d.)	187.8 (392.6)
Substance use disorder diagnosis (%)	392 (80.9)
Median hospital admissions with a SMI diagnosis in 5 years prior to first custody (range)	4 (1–89)
Post-release support (%)	
Never received support post-release	116 (24.7)
Ever received community mental health support post-release	357 (73.7)
Ever received disability support post-release	96 (19.8)
Ever received a combination of community mental health and disability support post-release	85 (18.6)
Age at study commencement, years (%)	
18–24	173 (35.7)
25–29	82(16.9)
30–39	149 (30.8)
40–49	62 (12.8)
50+	18 (3.7)
Remoteness of area (%)^a^	
1 – Major cities	309 (63.8)
2 – Inner regional	115 (23.8)
3 – Outer regional	42 (8.7)
4 – Remote/very remote	17 (3.5)
Prior adult custody episodes, *n* (%)	
No prior	253 (52.3)
1–5	126 (26.0)
5–10	55 (11.4)
10+	50 (10.3)
Index of Relative Socio-economic Disadvantage, quintile (%)^b^	
First quintile (most disadvantaged)	134 (27.7)
Second quintile	118 (24.4)
Third quintile	107 (22.1)
Fourth quintile	83 (17.1)
Fifth quintile (least disadvantaged)	37 (7.6)

a. One missing value.

b. Five missing values.

### SMI diagnoses

Table 2 shows a count of ICD-10 SMI diagnoses recorded during a hospital admission in the 5 year period prior to the first recorded prison episode in the study period. Diagnosis categories in Table 2 are not exclusive, as individuals can have multiple hospital admissions where a SMI has been recorded. The most common SMI was schizophrenia (71.3%), followed unspecified nonorganic psychosis (29.8%), acute and transient psychotic disorders (21.3%) and schizoaffective disorders (21.3%).

**Table 2:** Proportion of the cohort to have received a SMI ICD10 diagnosis in the 5 years prior to incarceration

Diagnosis code	Study cohort (*n* = 484)
Schizophrenia (F20)	345 (71.3%)
Schizotypal disorder (F21)	*n* < 10
Persistent delusional disorders (F22)	58 (12%)
Acute and transient psychotic disorders (F23)	103 (21.3%)
Induced delusional disorder (F24)	*n* < 10
Schizoaffective disorders (F25)	103 (21.3%)
Other nonorganic psychotic disorders (F28)	12 (2.5%)
Unspecified nonorganic psychosis (F29)	144 (29.8%)
Manic episode (F30)	16 (3.3%)
Bipolar affective disorder (F31)	89 (18.4%)

### Cox proportional hazards regression

The Cox proportional hazards model is shown in Table 3. Controlling for all other variables in the model, when compared to those not receiving support post-release, a reduced hazard of reincarceration was found for those who had received community mental health support (hazard ratio [HR] = 0.58, 95% CI 0.49–0.69, *P* < 0.001) or a combination of community mental health and disability support (HR = 0.46, 95% CI 0.34–0.61, *P* < 0.001) post-release. Receiving disability support compared with receiving no support in a post-release period had no association with lower hazards of reincarceration (HR = 0.98, 95% CI 0.67–1.43, *P* = 0.901). An increase in the hazard of reincarceration was associated with having a substance use disorder diagnosis (HR = 2.01, CI 1.58–2.56, *P* < 0.001) and having previous custody episodes (1–5 previous adult custodial episodes: HR = 1.51, 95% CI 1.15–1.98, *P* = 0.003; 6–10 previous adult custodial episodes: HR = 2.36, CI 1.64–3.40, *P* < 0.001); 10+ adult custodial episodes: HR = 1.95, CI 1.35–2.84, *P* < 0.001) compared with no previous custodial episodes. An increase in hazard of reincarceration was associated with a diagnosis of acute and transient psychotic disorders (F23: HR = 1.46, CI 1.27–1.68, *P* = 0.003) and unspecified nonorganic psychosis (F29: HR = 1.53, CI 1.35–1.74, *P* < 0.001).

**Table 3:** Hazard ratios from Cox proportional hazards regression on time to reincarceration

	HR (95% CI)^a^	*P*
Sex – male	0.93 (0.69, 1.24)	0.500
Custody length, days	1.05 (1.00, 1.17)	0.255
Substance use disorder	2.01 (1.58, 2.56)	<0.001
Country of birth – Australia	1.30 (0.84, 2.01)	0.200
Age, years		
18–24	Ref.	–
25–29	1.14 (0.92, 1.41)	0.200
30–39	0.86 (0.67, 1.10)	0.200
40–49	0.79 (0.57, 1.10)	0.055
49+	0.62 (0.33, 1.17)	0.140
	Wald test χ^2^ = 25.80, *P* < 0.001	
Post-release support		
No support	Ref.	−
Community mental health support	0.58 (0.49, 0.69)	<0.001
Disability support	0.98 (0.67, 1.43)	0.901
Community mental health and disability support	0.46 (0.34, 0.61)	<0.001
	Wald test χ^2^ = 33.87, *P* < 0.001	
Previous custody episodes, *n*		
0	Ref.	–
1–5	1.51 (1.15, 1.98)	0.003
6–10	2.36 (1.64, 3.40)	<0.001
10+	1.95 (1.35, 2.84)	<0.001
	Wald test χ^2^ = 109.99, *P* < 0.001	
Index of Relative Socio-economic Disadvantage – quintile		
First quintile (most disadvantaged)	Ref.	–
Second quintile	0.78 (0.66, 0.92)	0.110
Third quintile	0.87 (0.73, 1.03)	0.377
Fourth quintile	1.03 (0.86, 1.23)	0.867
Fifth quintile (least disadvantaged)	0.62 (0.46, 0.82)	0.063
	Wald test χ^2^ = 21.88, *P* < 0.001	
Remoteness of area		
1 – Major cities	Ref.	
2 – Inner regional	0.79 (0.67, 0.93)	0.104
3 – Outer regional	1.11 (0.89, 1.37)	0.628
4 – Remote/very remote	1.43 (1.06, 1.93)	0.312
	Wald test = χ^2^ = 8.34, *P* = 0.079	
ICD-10 SMI diagnosis		
Schizophrenia (F20)	1.22 (1.06, 1.50)	0.087
Schizotypal disorder (F21)	1.21 (0.80, 1.87)	0.673
Persistent delusional disorders (F22)	1.35 (1.27, 1.68)	0.067
Acute and transient psychotic disorders (F23)	1.46 (1.27, 1.68)	0.003
Induced delusional disorder (F24)	5.34 (0.84, 5.67)	0.087
Schizoaffective disorders (F25)	1.19 (1.03, 1.37)	0.192
Other nonorganic psychotic disorders (F28)	1.63 (1.24, 2.15)	0.117
Unspecified nonorganic psychosis (F29)	1.53 (1.35, 1.74)	<0.001
Manic episode (F30)	1.02 (0.74, 1.14)	0.938
Bipolar affective disorder (F31)	1.31 (1.12, 1.54)	0.060

Likelihood ratio test (279.8) = 1728, *P* < 0.001.

HR, hazard ratio.

a. Model includes Indigenous status as a control variable, results omitted.

## Discussion

### Main findings

This study examined the impact of provision of post-release disability and community mental health support on rates of reincarceration for a cohort with identified intellectual disability and SMI diagnosis. Our findings indicate that receiving community mental health support, or a combination of community mental health and disability support, is associated with a significantly lower rate of subsequent reincarceration for prisoners with intellectual disability and SMI. Receiving community mental health support post-release was associated with a 42% lower reincarceration rate, whereas receiving a combination of community mental health and disability support was associated with a 54% lower reincarceration rate. Increases in the hazard of reincarceration were associated with having a substance use disorder diagnosis, previous custody episodes, a diagnosis of acute and transient psychotic disorders, or a diagnosis of unspecified nonorganic psychosis.

### Community mental health, disability support and reincarceration

In this cohort study, contact with community mental health post-release from adult custody was associated with a lower hazard of reincarceration for prisoners with intellectual disability and a history of SMI. The greatest reduction in hazard of reincarceration was associated with those who received a combination of disability and community mental health support. The need for targeted support for prisoners with intellectual disability and specific mental and behavioural disorders is further emphasised by the increased hazard of reincarceration associated with particular psychiatric diagnoses, including acute and transient psychotic disorders and unspecified nonorganic psychosis. An increase in the hazard of reincarceration was also associated with a higher number of previous adult custodial episodes and a previous diagnosis of substance use disorder. Well-resourced post-release support services for prisoners with intellectual disability and SMI may help in addressing the high rates of incarceration and reincarceration for this group.

Offenders with intellectual disability often experience a complex mix of intellectual disability, mental illness, other developmental disorders, personality disorders, substance misuse and physical disorders.^21^ Overall, the lower hazard of reincarceration associated with post-release support found in this study reinforces prior studies showing that prisoners with intellectual disability experience better outcomes in relation to reincarceration when adequately supported post-release from prison.^13,22^ The finding of the lowest hazard of reincarceration for those who received a combination of disability and community mental health support highlights the value of addressing needs across a range of domains to reduce reoffending. Provision of disability support alone showed no effect on reincarceration, highlighting the importance of multidimensional models which address complex support needs for prisoners with intellectual disability and SMI.

The higher hazards of reincarceration associated with previous custodial episodes found in this study reinforce previous work linking past incarceration with the risk of future incarceration.^23^ This finding suggests that to address the cyclical nature of incarceration experienced by prisoners with intellectual disability,^24^ connection to support at a first custodial experience may alleviate some of the identified risk factors for reincarceration, including accommodation, income and employment, as well as linking individuals to community support services aimed at addressing longer-term risk factors for recidivism such as alcohol and drug dependence and mental illness.^13,14^

In the addition to issues related to cognition and mental health, prisoners with intellectual disability in this study also experienced problematic alcohol and substance use. This study found that 80% of the cohort received a diagnosis of mental and behavioural disorders owing to psychoactive substance use in the 5 years before the first custodial episode. The high rates of alcohol- and drug-related diagnoses found in this study's cohort reflect previous work indicating an overrepresentation of substance misuse in individuals identified with SMI^25^ and intellectual disability^26^ and an association with an increase in hazard of reincarceration.^27^ Importantly, studies have previously established that prisoners with intellectual disability receive less support for substance use disorders in prison than the general prisoner population^28^ and experience higher dropout rates from alcohol and drug support programmes.^29^ Our findings therefore indicate the potential value of additional specialised support for drug and alcohol comorbidities in this group.

### Implications

Despite differences across countries in service provision and legal approaches to offenders with intellectual disability, commonalities in studies of intellectual disability and the criminal justice system internationally include the enduring issues of overrepresentation of individuals with intellectual disability in prison,^4^ higher rates of reoffending^8–10^ and higher rates of mental illness.^1^ Our findings have important implications for the development and implementation of post-release support for prisoners with intellectual disability and a history of SMI. There is a distinct need in the NSW context for consistent approaches to the diagnosis of mental illness and identification of intellectual disability, which are both gateways to relevant support. Identification of intellectual disability and mental illness is required at the earliest points of contact with the criminal justice system. This can include the use of screening questionnaires at prisoner reception,^29^ with further and more detailed assessment when indicated. Mobilisation of appropriate coordinated supports and capacity to share information about support needs across agencies would potentially assist in the implementation of strategies to reduce offending and provide alternatives to reincarceration for this group. An integrated case management system may also assist by linking people in prison to individualised service provision pre-release to ensure continuity of care once in the community. Post-release, there is a need for enhanced, intensive and integrated specialist intellectual disability and community mental health services^1^ with links to supports in the justice, community and welfare, housing and health^30,31^ services. Finally, our findings point to the value of routinely linking data-sets across multiple agencies and jurisdictions to enable insights which may improve outcomes for those with complex support needs.

### Strengths and limitations

Key strengths of the linked administrative data used in this study include both the near-population-size sample of the intellectual disability population in NSW and the linking of health services, disability and community mental health support services data with data from corrective services in accordance with all ethical, legal, privacy and confidentiality requirements.^32^ There were several limitations to our work and its interpretation. It is likely that some people with intellectual disability in custody are never identified particularly, when they choose not to identify as having a disability. It is likely that not all offenders are identified in the linked data-sets or have been correctly diagnosed, especially where behaviour could be interpreted as meeting criteria for other diagnostic categories. Given the high proportion of the study cohort having a diagnosed substance use disorder, data related to substance use treatment support services would be a valuable addition to this study. However, it is likely that the inclusion of such data in this study would not have affected the results substantially, as people with intellectual disability receive fewer supports for substance use disorder both in and out of prison. The absence of individual level socioeconomic status in this study limits interpretation at the individual level. However, our results showed face validity, as people who lived in a more disadvantaged area are more likely to be reincarcerated than people who lived in a less disadvantaged area. Our analysis focussed on clinical contact with community mental health services. We did not have access to treatment data such as antipsychotic or other psychotropic medication use. In addition, data from general practice visits were not available for the present analysis. The aggregated nature of the DSMDS data does not allow for accurate appraisal of the timing of service delivery or of which particular support services were delivered, completed or prematurely ended. Incremental changes in the provision of disability and mental health support are likely to have occurred over the study period, but we could not account for such changes in this study.

## Data Availability

The uploading of these data to a public repository and/or release of these data to other researchers is prohibited by the ethical approval governing this project.
